# The subchondral bone healing after fixation of an osteochondral talar defect is superior in comparison with microfracture

**DOI:** 10.1007/s00167-017-4654-z

**Published:** 2017-07-27

**Authors:** Mikel L. Reilingh, Kaj T. A. Lambers, Jari Dahmen, Kim T. M. Opdam, Gino M. M. J. Kerkhoffs

**Affiliations:** 1 Department of Orthopedic Surgery, Academic Medical Center, University of Amsterdam, Meibergdreef 9, 1105 AZ Amsterdam, The Netherlands; 2 Academic Center for Evidence Based Sports Medicine (ACES), Meibergdreef 9, 1105 AZ Amsterdam, The Netherlands; 3 Amsterdam Collaboration for Health and Safety in Sports (ACHSS), Meibergdreef 9, 1105 AZ Amsterdam, The Netherlands

**Keywords:** Ankle, Osteochondral defects, Arthroscopy, Microfracture, Bone marrow stimulation, Fixation, Lift, drill, fill and fix (LDFF)

## Abstract

**Purpose:**

Arthroscopic bone marrow stimulation (BMS) has been considered the primary surgical treatment for osteochondral defects (OCDs) of the talus. However, fixation has been considered as a good alternative. Recently, a new arthroscopic fixation technique was described: the lift, drill, fill and fix procedure (LDFF). The purpose of this study was to evaluate the clinical and radiological results between arthroscopic LDFF and arthroscopic BMS in primary fixable talar OCDs at 1-year follow-up.

**Methods:**

In a prospective comparative study, 14 patients were treated with arthroscopic BMS and 14 patients with arthroscopic LDFF. Pre- and postoperative clinical assessment included the American Orthopaedic Foot and Ankle Society (AOFAS) score and the numeric rating scales (NRSs) of pain at rest and running. Additionally, the level of the subchondral plate (flush or depressed) was analysed on the 1 year postoperative computed tomography scans.

**Results:**

No significant differences in the AOFAS and NRS pain at rest and running were found between both groups at 1-year follow-up. After LDFF the level of the subchondral bone plate was flush in 10 patients and after BMS in three patients (*p* = 0.02).

**Conclusion:**

No clinical differences were found between arthroscopic LDFF and arthroscopic BMS in the treatment of talar OCDs at 1-year follow-up. However, the subchondral bone plate restores significantly superior after arthroscopic LDFF compared to arthroscopic BMS. It may therefore give less progression of ankle osteoarthritis in the future with a thus potential better long-term outcome.

**Level of evidence:**

III.

## Introduction

Osteochondral defects (OCDs) of the talus often have a severe impact on the quality of life of patients [25]. Currently, arthroscopic bone marrow stimulation (BMS) has been considered the primary surgical treatment for chronic OCDs up to 15 mm. This preference is based on the ease of execution of the technique, the low complication rate and high success rates reported in the literature [30, 31]. However, BMS does not aim at the preservation of a hyaline cartilage layer, but rather promotes the formation of fibrocartilage which decreases in quality over time and shows inferior wear characteristics [12, 13, 23]. Furthermore, after debridement and bone marrow stimulation the subchondral bone plate is often irregular and depressed [19]. These factors might be the reason why progression of ankle osteoarthritis is seen in 33–34% of the patients at long-term follow-up [4, 16, 24].

Recently, a new arthroscopic fixation technique for chronic primary talar OCDs was described: the lift, drill, fill and fix procedure (LDFF) [7]. The assumed theoretical advantages of this technique are the restoration of the subchondral bone plate and the preservation of hyaline cartilage. Promising clinical and radiological results were found in the first seven patients at 1-year follow-up. However, at present, no comparative study has been conducted between LDFF and BMS in primary fixable talar OCDs. Consequently, the aim of this study was to evaluate the clinical and radiological results between arthroscopic LDFF and arthroscopic BMS in primary fixable talar OCDs at 1-year follow-up.

## Materials and methods

This study was approved by the local medical ethics committee at the University of Amsterdam with reference number MEC 08/326 and performed in accordance with the current ethical standards (Declaration of Helsinki).

The study included patients with a symptomatic fixable primary talar OCD with a diameter >10 mm (in three dimensions) as measured on computed tomography (CT) scans. Fixable defects were defined as type II–IV, based on the Berndt and Harty classification [1]. Exclusion criteria were open physis of the distal tibia, ankle osteoarthritis grade II or III [26], concomitant OCD of the tibia, ankle fracture within 6 months before treatment of the OCD, surgical treatment of the index ankle performed within 1 year before treatment of the OCD, concomitant painful or disabling disease of the lower limb and rheumatoid arthritis.

### Population

As of 2013, we have prospectively recorded all patients undergoing an arthroscopic LDFF procedure [7]. For the control group (arthroscopic BMS), we used data from a randomized controlled trial (RCT) investigating pulsed electromagnetic fields (PEMF) after arthroscopic debridement and BMS [20]. Both the PEMF treatment and the placebo group were included in the arthroscopic BMS control group of the present study, as neither functional nor radiological differences between the groups were found in the previous trial. Patients were retrospectively selected to the BMS control group if their lesion could be defined as a fixable defect.

### Operative technique

#### Arthroscopic LDFF

All arthroscopic LDFF procedures were performed using a standardized technique by the senior author (GK) [7]. Anteromedial and anterolateral portals were created with the ankle in full dorsiflexion. The OCD was identified with a probe by moving the ankle in full plantar flexion. Subsequent to this, an osteochondral flap was created with use of a beaver knife and lifted with a chisel. The bone flake of the osteochondral fragment as well as the osteosclerotic area of the bed was drilled with the use of a K-wire and a shaver blade. Cancellous bone was harvested from the distal tibia and transported into the defect until there was sufficient substantial filling. Finally, the osteochondral flap was fixed with an absorbable bio-compression screw(s) (Arthrex Inc, Naples, USA) or/and a chondral dart(s) (Arthrex Inc, Naples, USA).

Postoperatively, a short-leg non-weight-bearing cast was applied for 4 weeks. After these 4 weeks, the foot was placed in a short-leg walking cast in neutral flexion position and neutral hindfoot position, with full weight bearing allowed. At 8 weeks postoperatively, the cast was removed. Physical therapy was prescribed to assist in functional recovery and extend to full weight bearing in approximately 2 weeks [7].

#### Arthroscopic BMS

All arthroscopic BMS procedures were performed using a standardized technique by the senior author (GK) [20]. Like in the LDFF technique, an anteromedial and an anterolateral portal was created. After identification of the OCD, all unstable bone and cartilage were removed with a curette and bone cutter shaver. This was followed by perforation with a microfracture awl, with intervals of approximately 3 mm. At the end of the procedure, a pressure bandage was applied.

Postoperative management consisted of a protocol-based rehabilitation programme, guided by a physiotherapist. Partial (eggshell) weight bearing on crutches was allowed as tolerated and progressed to full weight bearing over a period of 6 weeks. During this 6-week period, active non-weight-bearing and partial weight-bearing sagittal range of motion exercises were encouraged [27].

### Outcome assessment

Clinical outcome was assessed by means of numeric rating scales (NRSs) for pain (at rest and running) and the American Orthopaedic Foot and Ankle Society (AOFAS) ankle-hindfoot score [5, 6, 8]. These questionnaires were evaluated preoperatively and at 1 year postoperatively. The NRS is an 11-point scale, representing the spectrum of no pain (0 points) to the worst pain imaginable (10 points) [5]. The AOFAS is a 100-point score, with a subjective and an objective component, which devotes 40 points to pain, 50 to function and 10 to alignment [6, 8].

### Imaging

Computed tomography (CT) scans of the affected ankle were obtained preoperatively and at 1 year postoperatively. The scanning protocol involved “ultra-high-resolution” axial slices with an increment of 0.3 mm and a thickness of 0.6 mm, and multi-planar coronal and sagittal reconstructions of 1.0 mm [20]. CT scanning has been proven to be accurate in the detection and follow-up of OCDs of the talus, regarding location and extent as well as healing of the defect [14, 20, 28, 32].

On the preoperative CT scans, we graded the talar OCDs according to the modified Berndt and Harty classification [1, 22] and evaluated the OCD size by measuring the largest diameter (mm) in the anterior–posterior direction, medial–lateral direction and depth.

The level of the subchondral plate (flush or depressed) was analysed on the 1 year postoperative CT scans. Reilingh et al. [20] reported a good reliability in the measurements of the subchondral bone plate on CT scans. Furthermore, the union rate was evaluated on the postoperative CT scans after the LDFF procedure.

### Statistical analysis

Statistical analyses were conducted with Statistical Packages for Social Sciences (SPSS 23.0 Inc, Chicago, IL, USA) software. Continues data are presented as means with standard deviations or as medians with interquartile ranges (IQRs), depending on their distribution (normal or skewed). Comparison of the clinical outcome between groups was performed by the Student’s *t* test on normal distribution and the Mann–Whitney *U* test on skewed distribution. Additionally, the scale score differences between baseline and 1-year outcome assessment within each treatment group were analysed by using the paired *t* test on normal distribution and Wilcoxon signed-rank test on skewed distribution. The CT findings were analysed using the Chi-square test.

## Results

Out of our previous cohort [20], 14 patients were included who were treated with arthroscopic BMS in case of the presence of a fixable talar OCD. To create a similar and comparable cohort, we therefore only included the first 14 patients who were treated with arthroscopic LDDF. Both groups completed all questionnaires and the CT follow-up at 1 year postoperatively. The baseline characteristics are presented in Table 1. Patients in the LDFF group were significantly younger (*p* < 0.01) and had a lower body mass index (BMI) (*p* < 0.01). There was no significant difference in OCD classification or size of the lesion. Fixation was performed in nine cases with bio-compression screw(s), in three cases with chondral dart(s) and in two cases with a combination of both.

**Table 1:** Baseline characteristics of the patients

	LDFF, *n* = 14	BMS, *n* = 14	*p* value
Age (years), median (IQR)	17 (16–18)	23 (20–30)	<0.01
Gender, *n* (% male)	5 (36)	5 (36)	n.s.
BMI, mean (SD)	22 (3)	27 (4)	<0.01
Included side, *n* (% right)	10 (71)	12 (86)	n.s.
OCD size, mean (SD)			
Anteroposterior (mm)	13 (2)	12 (3)	n.s.
Medial–lateral (mm)	9 (2)	9 (2)	n.s.
Superior–inferior (mm)	6 (3)	5 (2)	n.s.
OCD classification, *n* (%)			
Partially fractured	2 (14)	2 (14)	n.s.
Completely undisplaced fracture	12 (86)	10 (72)	n.s.
Displaced fracture	0 (0)	2 (14)	n.s.

*IQR* interquartile range, *SD* standard deviation, *n.s.* not significant

### Clinical results

**Fig. 1:**
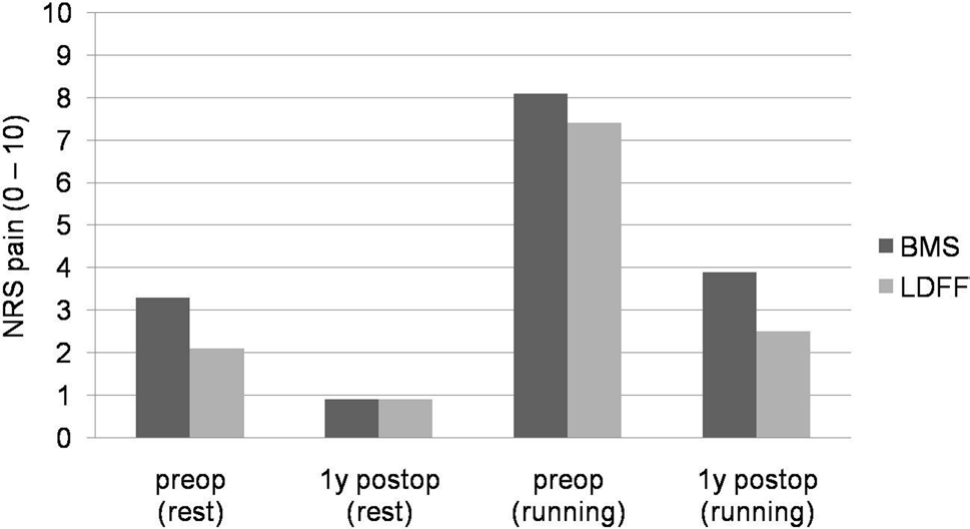
Graph showing the mean numeric rating scales (NRSs) for pain (at rest and when running) pre- and postoperatively. No significant differences were found between arthroscopic LDFF and arthroscopic BMS

**Fig. 2:**
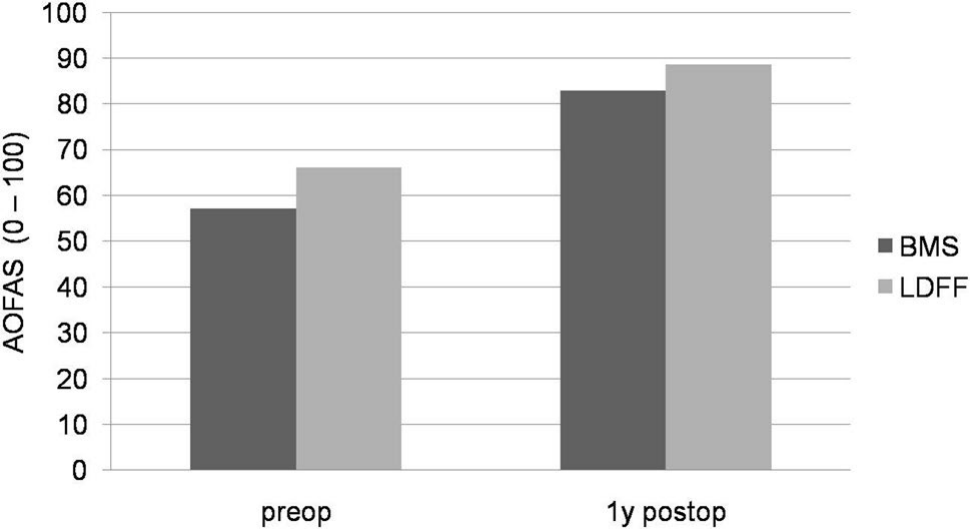
Graph showing the mean American Orthopaedic Foot and Ankle Society (AOFAS) ankle–hindfoot score pre- and postoperatively. No significant differences were found between arthroscopic LDFF and arthroscopic BMS

Both preoperatively and 1 year postoperatively, no significant differences in the AOFAS and NRS pain at rest and running were found between arthroscopic LDFF and arthroscopic BMS (Figs. 1, 2).

Within both treatment groups, the NRS pain and AOFAS improved significantly from preoperatively to 1 year postoperatively. After arthroscopic LDFF, the AOFAS significantly improved from 66 (SD 10.1) to 89 (SD 17.0) (*p* = 0.004). The NRS pain at rest significantly improved from 2.1 (SD 1.8) to 0.9 (SD 1.3) (*p* = 0.043), and NRS pain when running improved from 7.4 (SD 1.9) to 2.5 (SD 3.1) (*p* = 0.004) (Figs. 1, 2). After arthroscopic BMS, the AOFAS significantly improved from 57.1 (SD 13.6) to 83 (SD 15.9) (*p* < 0.001). The NRS pain at rest significantly improved from 3.3 (SD 1.5) to 0.9 (SD 1.7) (*p* = 0.001), and NRS pain when running improved from 8.1 (SD 1.7) to 3.9 (SD 2.8) (*p* < 0.001) (Figs. 1, 2).

### Radiological results

**Fig. 3:**
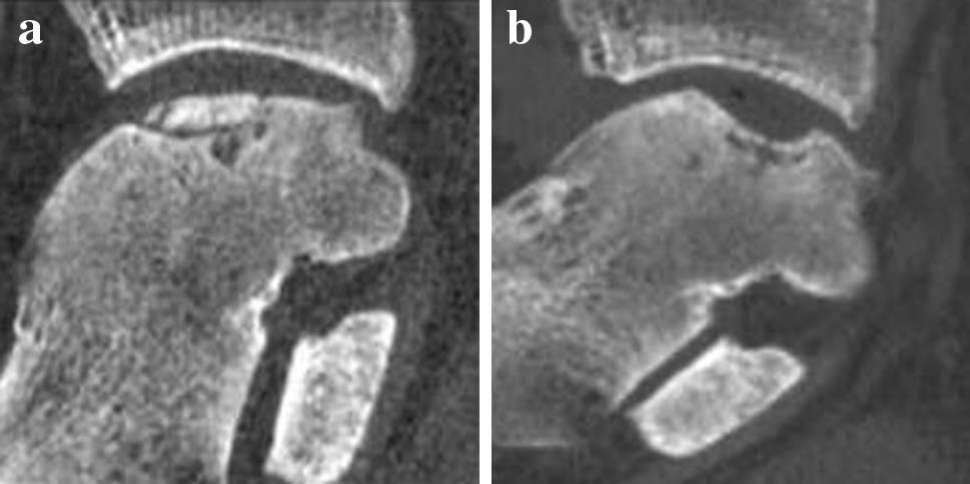
**a** Preoperative sagittal CT of a medial osteochondral talar defect of a right ankle. **b** Postoperative sagittal CT of the same ankle after arthroscopic debridement and bone marrow stimulation (BMS) at 1-year follow-up

**Fig. 4:**
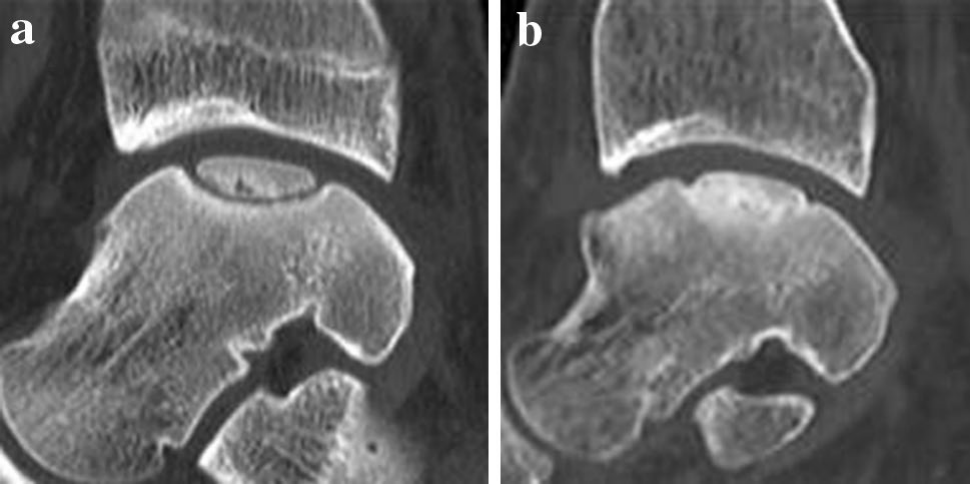
**a** Preoperative sagittal CT of a medial osteochondral talar defect of a right ankle. **b** Postoperative sagittal CT of the same ankle after arthroscopic lift, drill, fill, and fix (LDFF) at 1-year follow-up

A significant difference (*p* = 0.02) was found in the healing of the subchondral bone plate between both groups. After arthroscopic BMS, a depressed subchondral bone plate was observed in 11 patients and three patients had a flush subchondral bone plate (Fig. 3), while after arthroscopic LDFF, a depressed subchondral bone plate was found in four patients and a flush subchondral bone plate in 10 patients (Fig. 4).

Union of the osteochondral fragment was found in nine patients after arthroscopic LDFF.

### Complications

No serious adverse event occurred in either groups. One patient had prolonged wound leakage during the first week after arthroscopic BMS. No complications were reported after arthroscopic LDFF.

## Discussion

The most important findings of the present comparative study were that no clinical differences were found between arthroscopic LDFF and arthroscopic BMS at 1-year follow-up. However, the subchondral bone plate restores significantly better after LDFF in comparison with BMS. Union of the fragment was found in nine out of 14 patients, but was not associated with a better outcome. This could be explained because a non-united fragment was stabilized by scar tissue and was no longer an intra-articular loose body.

The healing of the subchondral bone plate is important in the surgical treatment of OCDs. Research has indicated that an irregular subchondral bone plate has a negative effect on cartilage repair and thus plays an important role in the development of osteoarthritis [9, 10, 12, 15, 16]. Progression of ankle osteoarthritis is seen in 33–34% of the patients following arthroscopic debridement and BMS at long-term follow-up [4, 16, 24]. Although the long-term clinical and radiological outcomes of the arthroscopic LDFF procedure have not been researched yet, it is postulated that progression of ankle osteoarthritis is less than in patients treated with BMS because the subchondral bone plate restores more in accordance with the normal congruency of the ankle. 78–100% of the patients were regarded clinically successful in case series describing open fixation of talar OCDs at mid-term follow-up [11, 18, 21]. Furthermore, in an earlier study we found no progression of osteoarthritis after open fixation of talar OCDs in children at mid-term follow-up [18].

To the best of our knowledge, this is the first prospective comparative study investigating the clinical and radiological changes between arthroscopic LDFF and arthroscopic BMS. Strengths of this study include the prospective methodology and the complete radiological and clinical follow-up. Furthermore, the defect size was equally distributed between both groups. This is important because larger defects are associated with poorer outcomes [2, 3, 17]. Limitations include the lack of long-term follow-up and power analysis. Furthermore, BMI was significantly lower and patients were significantly younger in the LDFF group. These factors are associated with superior outcomes [18, 20]. However, it must be noted that none of the patients were classified as obese according to the WHO standards [29]. Furthermore, only skeletally mature patients were included in this study.

Based on the radiological results, fixation of a talar OCD with a bony fragment should be considered as the primary surgical treatment.

## Conclusion

No clinical differences were found between arthroscopic debridement and BMS and arthroscopic LDFF in the treatment of osteochondral talar defects at 1-year follow-up. However, the subchondral bone plate restores significantly superior after arthroscopic LDFF compared to arthroscopic BMS.
